# Age-Related Immune Responses and Long-Term Immunity in Adult Sheep and Goats Following Vaccination with the Nigeria 75/1 Live Attenuated PPR Vaccine

**DOI:** 10.3390/vetsci13050433

**Published:** 2026-04-28

**Authors:** Yerbol Bulatov, Abdurakhman Ussembay, Zhanat Amanova, Zhanna Sametova, Zhanat Kondibayeva, Ruslan Abitayev, Sholpan Turyskeldi, Kuandyk Zhugunissov, Zhumagali Koshemetov, Aslan Kerimbayev, Felix Njeumi, Dariya Toktyrova

**Affiliations:** 1 Research Institute for Biological Safety Problems, National Holding “QazBioPharm”, Gvardeyskiy 080409, Kazakhstan; ye.bulatov@biosafety.kz (Y.B.); a.ussenbay@biosafety.kz (A.U.); zh.amanova@biosafety.kz (Z.A.); zh.sametova@biosafety.kz (Z.S.); zh.kondybaeva@biosafety.kz (Z.K.); r.abitaev@biosafety.kz (R.A.); sh.smankizi@biosafety.kz (S.T.); k.zhugunisov@biosafety.kz (K.Z.); zh.koshemetov@biosafety.kz (Z.K.); a.kerimbayev@biosafety.kz (A.K.); 2 Food and Agriculture Organisation of the United Nations (FAO), Viale delle Terme di Caracalla, 00153 Rome, Italy; felix.njeumi@fao.org

**Keywords:** peste des petits ruminants, attenuated vaccine, Nigeria 75/1 strain, immunogenicity, sheep and goats

## Abstract

In 2023, a highly immunogenic live attenuated vaccine based on the Nigeria 75/1 strain was introduced in Kazakhstan to provide protection against PPR. This study presents the results of a three-year animal trial evaluating the vaccine’s efficacy, safety, and immunogenicity. The novelty of this study lies in the long-term (up to 36 months) evaluation of protective immunity in adult animals, as well as in the comparative analysis of immune responses across different age groups and the assessment of viral suppression following challenge infection. Sheep and goats of different age groups were included, including lambs and kids aged 1.5 and 3 months, as well as adult animals aged 2–3 years. The vaccine was well tolerated following a single immunization, and no clinically significant adverse effects were observed in vaccinated animals, apart from only mild transient local reactions. A strong humoral (IgG) response to PPRV antigens was detected in all groups, with the highest antibody titers observed in young animals. Seroconversion was detected in 100% of vaccinated animals by day 21 post-vaccination. Long-term protective immunity (at least 36 months) was demonstrated in adult animals, whereas in young animals early protection was confirmed at 21 days post-vaccination along with subsequent humoral immune dynamics following a single immunization with a 1.0 mL dose of the vaccine (Nigeria 75/1 strain, titer 10^3.0^ TCID_50_/mL). These findings indicate that the vaccine is well tolerated, highly immunogenic, and provides sustained protection in adult animals while inducing early immune responses in young animals.

## Simple Summary

Peste des petits ruminants is a highly contagious viral disease that causes severe economic losses in sheep and goat production worldwide. Vaccination remains the most effective strategy for controlling and preventing the disease. In Kazakhstan, a live attenuated vaccine based on the Nigeria 75/1 strain was introduced to improve protection against PPR in small ruminants. This study summarizes the results of a three-year animal trial evaluating the safety, immunogenicity, and duration of immunity induced by this vaccine. Sheep and goats of different age groups, including lambs and kids aged 1.5 and 3 months, as well as adult animals aged 2–3 years, were included in the study. Following a single vaccination, no clinically significant adverse effects were observed, confirming that the vaccine was well tolerated under the conditions of this study. All vaccinated animals developed antibodies against the PPRV. Long-term protective immunity was demonstrated in adult animals throughout the 36-month observation period, whereas in young animals early immune responses and subsequent humoral dynamics were documented. These results indicate that a single dose of the Nigeria 75/1 live attenuated vaccine provides sustained protection in adult animals and induces early immune responses in young sheep and goats.

## 1. Introduction

Peste des petits ruminants (PPR) is an economically significant, acute or subacute, highly contagious viral disease affecting sheep, goats, and certain wild ruminants [1]. The disease is characterized by high fever, nasal discharge, pneumonia, stomatitis and inflammation of the gastrointestinal tract [2]. The causative agent is the Peste des petits ruminants virus (PPRV), an RNA virus belonging to the genus *Morbillivirus* of the family *Paramyxoviridae* and the order *Mononegavirales* [3]. PPRV shares antigenic similarities with other morbilliviruses, including canine distemper virus (CDV), the eradicated rinderpest virus (RPV) and measles virus (MV). Some morbilliviruses (e.g., CDV and MV) are known to cross species barriers and infect atypical hosts [4]. Although PPR primarily affects goats and sheep, sporadic infections have been reported in wild ruminants [5], buffaloes [6], camels [7] and other atypical hosts such as pigs [8] in endemic regions. However, apart from sheep and goats, other species generally do not play a significant epidemiological role, as they do not shed the virus as efficiently as small ruminants [9]. PPRV is transmitted through close contact between susceptible and infected animals—via aerosols and various secretions (conjunctival, nasal, oral, and rectal). The virus may also spread through contaminated materials, such as water, feeding troughs, and bedding. Due to its pronounced thermolability and rapid environmental inactivation, PPRV transmission predominantly occurs through direct contact between infected and susceptible animals. [10]. Animals that recover from PPR develop lifelong immunity [11,12]. Due to the immunosuppressive nature of the virus, secondary infections frequently occur, complicating clinical diagnosis [13]. Outbreaks of PPR are generally more severe in goats than in sheep, and the disease tends to be more acute in newborn and young animals than in adults [14]. In primary outbreaks, morbidity and mortality rates can reach up to 90% and 100%, respectively. Therefore, PPR is listed by the WOAH as a notifiable transboundary animal disease of major importance [15].

Despite existing control measures, including legal frameworks, available vaccines, and diagnostic tools, peste des petits ruminants (PPR) remains a serious epizootic threat [16]. In the Republic of Kazakhstan, the disease is not officially recognized as endemic. However, some studies suggest the possible silent circulation of the virus within the country. According to Lundervold et al. [17], antibodies to PPRV were first detected in 1997–1998 in Central Kazakhstan (Dzhezkazgan Region, now Ulytau Region) in a limited number of sheep, goats, and cattle. Based on available data, an outbreak of an unidentified infectious disease was reported in southern Kazakhstan in 2003, affecting sheep and goats. Clinical, epizootiological and serological investigations confirmed the causative agent as a highly pathogenic PPR virus, which was isolated and designated as “Kentau-7” [18]. From 2003 to 2014, Kazakhstan did not report any new PPR cases to the WOAH. Molecular characterization of PPR virus isolates obtained in 2014 revealed a high genetic similarity (99.5–99.7%) to strains circulating in Heilongjiang Province, China [19]. Previously, PPR outbreaks were reported in Tibet (2007–2010) and in Xinjiang, near the Kazakh border (December 2013). In total, during 2013 and the first half of 2014, 273 PPR outbreaks were reported in China, with 33.1 thousand cases, over 60.1 thousand animals culled and destroyed, and 74.9 thousand sheep and goats located in outbreak and at-risk zones [20]. The high genetic identity between the Kazakhstani isolates and contemporaneously circulating Chinese strains suggests a potential transboundary transmission dynamic of the pathogen [21].

The southern regions of Kazakhstan, which are home to approximately 60% of the country’s small ruminant population, are considered high-risk areas for PPRV introduction, while the rest of the territory is classified as low-risk. Vaccination is implemented only in high-risk zones, covering between 12% and 42% of the small ruminant population from 2017 to 2021; however, post-vaccination assessment has not been conducted. Serological surveillance is performed twice a year, though with certain limitations. According to the Global Strategy for the Control and Eradication of PPR, the low-risk zone in Kazakhstan is classified as stage 4, while the high-risk zone is at stage 3. As of now, Kazakhstan remains among the countries without an official PPR status, according to WOAH [22,23]. Nevertheless, PPR continues to pose a significant transboundary epizootic risk [24].

This article presents the results of a preclinical (experimental) study with elements of controlled field trials conducted in Kazakhstan between 2021 and 2023, during which a highly immunogenic vaccine against PPR based on the attenuated Nigeria 75/1 strain was developed and introduced. The focus was on evaluating the long-term immunity and safety of the vaccine across different age groups, supporting Kazakhstan’s national PPR eradication program. Previous studies conducted within this program assessed antibody levels in vaccinated pregnant Kazakh fine-wool ewes and the duration of maternal immunity in their lambs [25]. Following the completion of these studies and in accordance with established regulatory procedures, the vaccine successfully underwent registration in the State Register of Veterinary Medicinal Products of the Republic of Kazakhstan (Reg. No. RK-VP-1-5168-23, 15 December 2023), based on confirmed safety and high efficacy demonstrated in clinical trials.

## 2. Materials and Methods

### 2.1. Animals

The experimental vaccine was tested on lambs and kids aged 1.5 and 3 months (body weight 5–8 kg and 20–25 kg, respectively), as well as on adult sheep and goats aged 2–3 years. Only animals that tested seronegative for PPRV antibodies and had not been previously vaccinated were selected for the study. Prior to the experiments, all animals were quarantined for two weeks and underwent daily rectal temperature monitoring, individual identification, clinical examination, and serological testing (ELISA) to confirm the absence of PPRV antibodies. During the experiments, special attention was paid to the animals’ health status, antibody dynamics, clinical signs, and the timing of immune response development. Each animal group was housed in a separate isolation unit. The vaccine was administered subcutaneously. Throughout the experimental period, rectal temperature and general health status were monitored, and serological tests were performed. All procedures involving animals were carried out in strict accordance with bioethical standards.

### 2.2. Vaccine Strain

An attenuated PPR virus strain, Nigeria 75/1 (GenBank: KY628761.1), with a biological activity of 6.08 ± 0.08 log TCID_50_/mL, was used for vaccine development and experimental studies. The vaccine strain was obtained from the Microorganism Collection Laboratory of the Research Institute for Biological Safety Problems (RIBSP, Gvardeiskiy, Kazakhstan).

The virus was cultivated and its biological and immunogenic activity evaluated using Vero (ATCC, CCL-81) cell culture. Dulbecco’s Modified Eagle Medium (DMEM) was used as both the growth and maintenance medium. The vaccine formulation included 5% peptone and 3% sucrose as stabilizers and was subjected to lyophilization via vacuum sublimation. The final lyophilized vaccine complies with cold chain requirements, with a shelf life of 12 months when stored at +2 to +8 °C. At +20 to +25 °C, the vaccine retains its immunogenic properties for up to 5 days, and at +35 to +37 °C—for up to 3 days.

### 2.3. Challenge Virus

A field strain of the PPR virus, “Kentau-7,” (IV lineage) was used as the challenge virus. This strain is deposited in the Microorganism Collection Laboratory of the Research Institute for Biological Safety Problems (RIBSP), Gvardeiskiy, Kazakhstan. It was originally isolated in 2003 from pathological material collected from a goat during a PPR outbreak in Kazakhstan. The virus was obtained through serial passage (seven passages) of the supernatant from infected organ tissues on primary lamb kidney cell culture [26].

### 2.4. Study Design

Prior to use, the lyophilized vaccine was reconstituted with sterile physiological saline to the original volume. A total of 126 animals (sheep and goats) were included in the experiment. The vaccine was tested on animals aged 1.5 months, 3–5 months and 2–3 years. Animals were randomly assigned to experimental and control groups using an online random number generator. Randomization was performed separately within each age category to ensure balanced distribution of sheep and goats across groups. No additional stratification by breed was applied.

Group allocation was performed prior to the start of the experiment. Clinical observations and laboratory analyses (including ELISA and virus neutralization test, VNT) were conducted by personnel not involved in group allocation. Laboratory analyses were performed with elements of blinding. Animals were randomly assigned to groups using an online random number generator:

Group I—1.5-month-old young animals (*n* = 24; 12 lambs and 12 kids): subcutaneous immunization with a single dose of the PPR vaccine (1.0 × 10^3.0^ TCID_50_/mL).

Group II—3-month-old young animals (*n* = 24; 12 lambs and 12 kids): subcutaneous immunization with a single dose of the PPR vaccine (1.0 × 10^3.0^ TCID_50_/mL).

Group III—Adults aged 2–3 years (*n* = 24; 12 sheep and 12 goats): subcutaneous immunization with a single dose of the PPR vaccine (1.0 × 10^3.0^ TCID_50_/mL).

Groups IV, V, and VI (control) consisted of unvaccinated animals and served as controls for the experimental groups I, II, and III, respectively (8 lambs and 8 kids in groups IV and V, and 8 sheep and 8 goats in group VI; total n = 48). These animals received 1.0 mL of sterile phosphate-buffered saline.

An additional group was formed to assess reactogenicity (Group VII), comprising 3 sheep and 3 goats. The animals were subcutaneously administered the vaccine into a hairless area (axillary region) at an increased dose of 1.0 × 10^5.0^ TCID_50_/mL to evaluate the safety and tolerability of high vaccine doses. The subcutaneous route was selected in accordance with standard vaccination protocols for live attenuated PPR vaccines, allowing controlled and reproducible administration. However, this route differs from natural infection pathways, which should be considered when interpreting protective mechanisms.

After vaccination, animals were monitored daily for 14 days for general condition, local reactions at the injection site, and rectal temperature. Immune responses were evaluated by measuring antibody dynamics using ELISA and VNT.

To evaluate the protective efficacy of the vaccine, animals were challenged with the virulent PPRV strain “Kentau-7.” For the challenge study, animals were subcutaneously inoculated in the subscapular region with 1.0 mL of viral suspension containing 1000 LD_50_/mL. Challenged animals were maintained in the organization’s isolation units under institutional biosafety procedures.

Following challenge, animals were observed for 14 days. Rectal temperature and clinical signs of PPR were recorded daily and assessed according to a previously described scoring system [27].

### 2.5. Determination of the Biological Activity of PPRV

The biological activity of the vaccine virus was determined by titration in Vero cell culture (48-well plates, TPP, Techno Plastic Products AG, Trasadingen, Switzerland). The titration procedure was performed as follows; serial tenfold dilutions of the virus-containing sample were prepared, ranging from 10^−1^ to 10^−7^, using DMEM maintenance medium. Each dilution was inoculated into four wells containing a monolayer of Vero cells, with 200 μL of suspension added per well. A separate plate with uninfected cell culture served as the negative control. Both infected and control plates were incubated at 37 °C in a 5% CO_2_ atmosphere, with medium replacement every 48 h.

Cytopathic effects (CPE) in infected cells were monitored daily using an inverted light microscope (Diavert Leitz, Wetzlar, Germany) under low magnification. Observations were conducted over 12–14 days. At the end of the observation period, the virus titer was calculated using the Reed and Muench method.

### 2.6. Serum Sample Collection

Blood samples from both experimental and control animals were collected into vacuum tubes containing a clot activator and separation gel on days 7, 14, and 21, and subsequently at 1, 3, 6, 9, 12, 15, 18, 21, 24, 27, 30, and 36 months post-vaccination. The samples were used to determine virus-neutralizing antibody (VNA) levels against PPRV via the virus neutralization test (VNT) and to quantify antibody levels by ELISA.

### 2.7. RNA Extraction and Molecular Genetic Analysis of PPRV

Swabs were collected daily from all animals in both experimental and control groups from day 1 to day 7, and additionally on days 10 and 14 post-vaccination. Swabs were placed in tubes containing 1.0 mL of viral transport medium (phosphate-buffered saline with 2% antibiotic solution). Viral RNA was extracted from swab samples using the commercial ID GeneTM Mag Fast Extraction Kit (QIAGEN, Hilden, Germany) according to the manufacturer’s instructions.

Detection of the viral genome was performed by reverse transcription followed by quantitative PCR (RT-PCR) using the ID Gene™ PPR Duplex kit (ID.vet, Grabels, France). Duplex kit (ID.vet, Grabels, France) on the Rotor-Gene Q thermal cycler (QIAGEN, Hilden, Germany), following the manufacturer’s instructions. Results were interpreted based on cycle threshold (Ct) values.

The RT-PCR protocol included the following steps (Table 1):

### 2.8. ELISA for the Detection of Antibodies Against PPRV

Serum samples from vaccinated and control animals were tested using a competitive enzyme-linked immunosorbent assay (cELISA) with the ID Screen^®^ PPR Competition (PPRC-4P) kit (ID.vet, Montpellier, France) according to the manufacturer’s instructions. For each sample, the signal-to-noise ratio (S/N%) was calculated. Values of S/N ≤ 50% were considered positive (presence of specific antibodies), S/N between 50–60% were considered doubtful and S/N > 60% were interpreted as negative.

### 2.9. Determination of VNA Titers by VNT

Virus-neutralizing antibodies (VNA) in serum were quantified using the virus neutralization test (VNT). The procedure was as follows: sera collected from vaccinated and control animals (sheep and goats) were twofold serially diluted in sterile Hank’s solution, from 1:2 to 1:128. Each serum dilution was mixed with an equal volume of PPRV (200 TCID_50_/mL) and incubated at 4 °C for 14–16 h. Prior to inoculation, the virus-serum mixtures were additionally incubated at 37 °C for 1–1.5 h. The resulting suspensions were then added to monolayers of Vero cells. Serum from unvaccinated, healthy sheep (lacking neutralizing antibodies) was used as negative controls. The infected cultures were incubated at 37 °C for up to 14 days (until the development of a pronounced cytopathic effect, CPE).

Results were interpreted based on the presence or absence of CPE. Virus-neutralizing activity was expressed as the neutralization index (NI), calculated as the difference between the logarithmic titers of the test serum and the control serum. An NI of ≥1.0 log was considered positive (indicating the presence of specific VNA). Each VNT assay included controls for serum cytotoxicity and for the specificity of viral CPE.

### 2.10. Bioethics

All animal experiments were conducted in accordance with the Law of the Republic of Kazakhstan № 97-VII (30 December 2021) on the Responsible Treatment of Animals, as well as other applicable regulations. The study protocols were approved by the Bioethics Oversight Committee of the Research Institute for Biological Safety Problems (approval protocol № 3101/14) prior to the commencement of the research. All institutional guidelines and standard operating procedures governing the care and use of experimental animals were strictly followed throughout the study.

### 2.11. Statistical Analysis

Statistical analyses included calculation of the arithmetic mean (M), standard deviation (σ), standard error of the mean (m), and confidence intervals. Virus titers were determined using the Reed-Muench method. The normality of data distribution was assessed and differences between groups were evaluated using two-way ANOVA (GraphPad Prism 8.4.3) or Student’s t-test, as applicable. Statistical significance was accepted at *p* ≤ 0.05. Vaccine efficacy between groups was additionally assessed using the one-sided Fisher’s exact test at α = 0.05.

## 3. Results

### 3.1. Clinical Condition of Animals After Immunization

Post-vaccination reactions in immunized animals remained within physiological limits throughout the 14-day observation period. No abnormal clinical signs, behavioral changes, or reduced appetite were observed.

**Figure 1:**
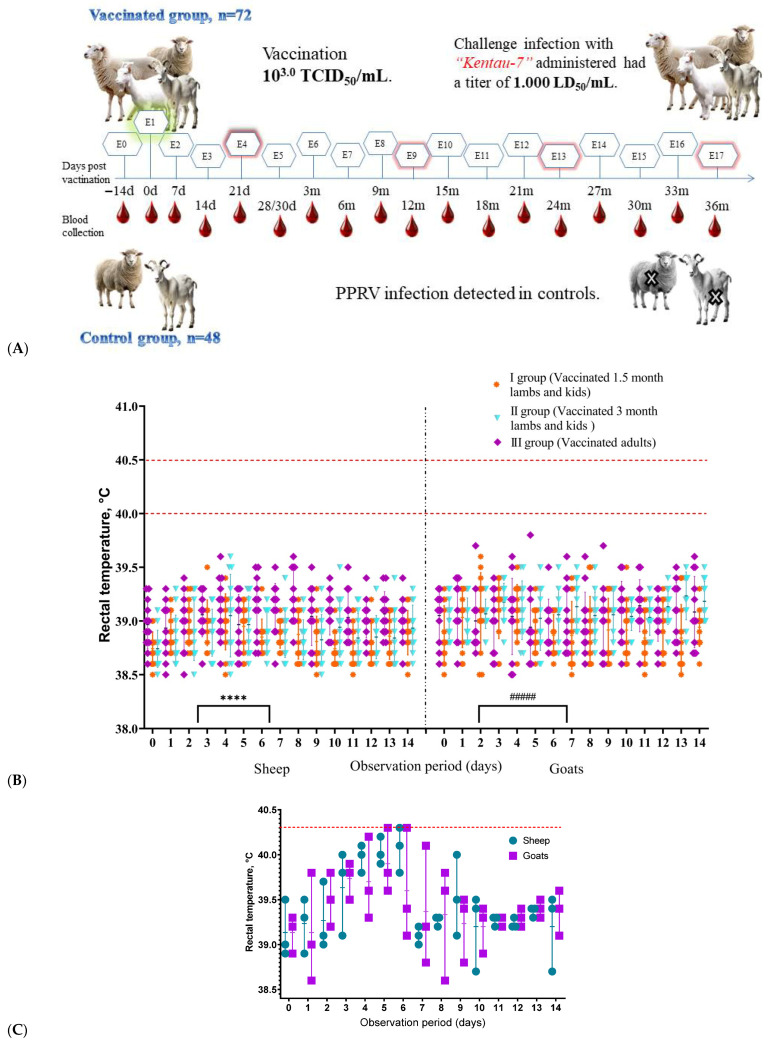
(**A**) Schematic representation of the study design; the green mark indicates the start day of the experimental work and red marks indicate the days of challenge with the virulent virus strain. (**B**) Dynamics of rectal temperature in sheep and goats during the evaluation of the immunogenicity and safety of the Nigeria 75/1 vaccine. (**C**) Rectal temperature data for six animals from Group VII that received a high vaccine dose. (* and #) denote local reactions observed in vaccinated sheep and goats post-immunization. The dashed line indicates the upper limit of normal body temperature. The red-highlighted text indicates the strain name.

Animals that received a high dose for the assessment of reactogenicity and vaccine safety also showed no clinical signs of disease or observable adverse effects (Figure 1C). It should be noted that the assessment of safety in this group was based on clinical observations, and no pathological or histopathological examinations were performed.

Local reactions were recorded in 9 out of 72 vaccinated animals (12.5%; 4 sheep and 5 goats; Figure 1B) and appeared as pinkish, irregularly rounded swellings approximately 0.4–0.6 cm in diameter and protruding 2.5–3.3 mm above the skin surface. These local swellings resolved spontaneously within 4–5 days and were not accompanied by an increase in body temperature.

Analysis of rectal temperature curves showed that some vaccinated animals had a slight transient increase in temperature between days 2 and 9. However, all values remained within the physiological range (38.5–39.8 °C) throughout the 14-day observation period (Figure 1C). These fluctuations were not associated with other clinical abnormalities and were considered to reflect normal physiological variation. Feed intake and general behavior in vaccinated animals remained comparable to those of the control group. Analysis of rectal temperature dynamics during the 14-day observation period revealed no significant differences between the vaccinated, control, and high-dose groups (two-way repeated-measures ANOVA, *p* > 0.05). Thus, no statistically significant temperature increase attributable to vaccination was observed.

### 3.2. Serological Monitoring of Post-Vaccination Immunity

**Figure 2:**
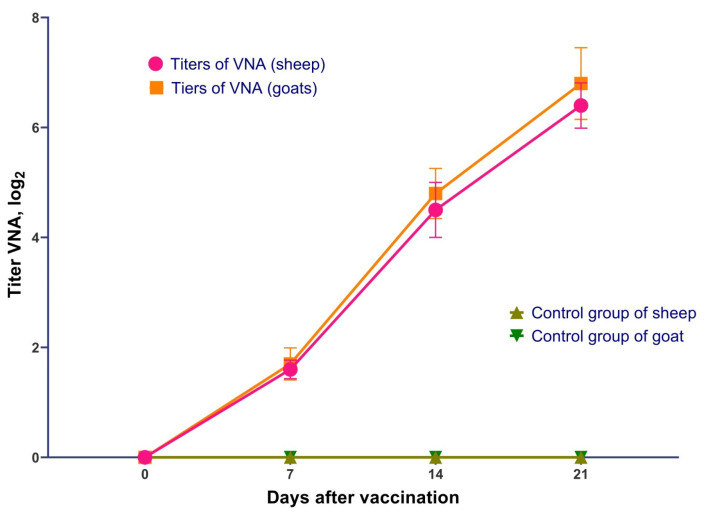
Dynamics of virus-neutralizing antibody (VNA) titers in sheep and goats following vaccination with the attenuated PPR vaccine. Note: the vaccinated group consisted of 72 animals, while the control group consisted of unvaccinated animals (*n* = 48).

As a result of serological studies, VNA against PPRV were detected in all groups of vaccinated sheep as early as day 7 post-vaccination, with an average titer of 1.6 log_2_. By day 14, the average VNA titer in sheep increased to 4.5 log_2_, reaching a maximum of 6.4 log_2_ by day 21. In vaccinated goats, VNA were also detected by day 7, with an average titer of 1.7 log_2_; on day 14, the average titer reached 4.8 log_2_, and the maximum value of 6.8 log_2_ was observed on day 21. Virus-neutralizing antibodies to the PPR virus were not detected in any of the animals belonging to the control groups. The VNA development dynamics in vaccinated sheep and goats are shown in Figure 2. In vaccinated sheep and goats, VNA titers increased significantly from day 7 to day 21 post-vaccination (*p* < 0.05), whereas no virus-neutralizing antibodies were detected in control animals throughout the observation period.

An additional analysis of sera from vaccinated animals using ELISA confirmed the absence of antibodies to PPRV prior to immunization (all samples had S/N ratios > 150%).

**Figure 3:**
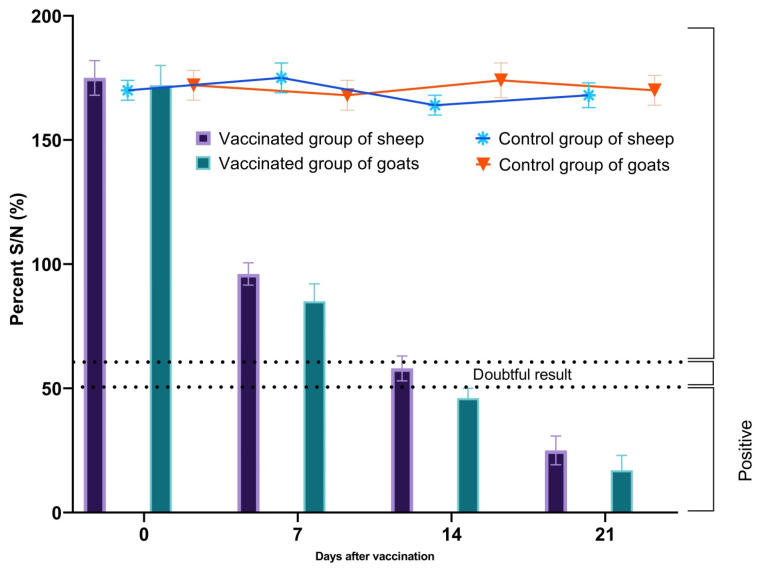
Evolution of the mean percentage of competitive inhibition (± standard deviation) in vaccinated and control sheep and goats following a single vaccination. Bar charts represent vaccinated animals, while line graphs represent control animals. Note: the vaccinated group consisted of 72 animals, while the control group consisted of unvaccinated animals (*n* = 48). S/N values: ≤50% were considered positive, 50% < S/N ≤60% were considered doubtful, and S/N >60% were considered negative.

By day 7 post-vaccination, the S/N percentage decreased to 96% in sheep and 85% in goats. Approximately 56% of vaccinated animals showed protective antibody levels (S/N ≤ 50%), while 28% still had titers above the threshold (S/N > 60%), and the remaining animals exhibited borderline results (50% < S/N ≤ 60%). However, by day 14, S/N values declined to 58% in sheep and 46% in goats, indicating protective immunity in about 95% of vaccinated animals. By day 21 post-vaccination, all vaccinated sheep and goats developed protective antibody levels, as the mean S/N ratios dropped to 25% and 17%, respectively (Figure 3). A significant decrease in S/N values was observed over time in vaccinated animals (*p* < 0.05), whereas control animals remained seronegative throughout the study period.

### 3.3. Duration of Immunity in Young Sheep and Goats

**Figure 4:**
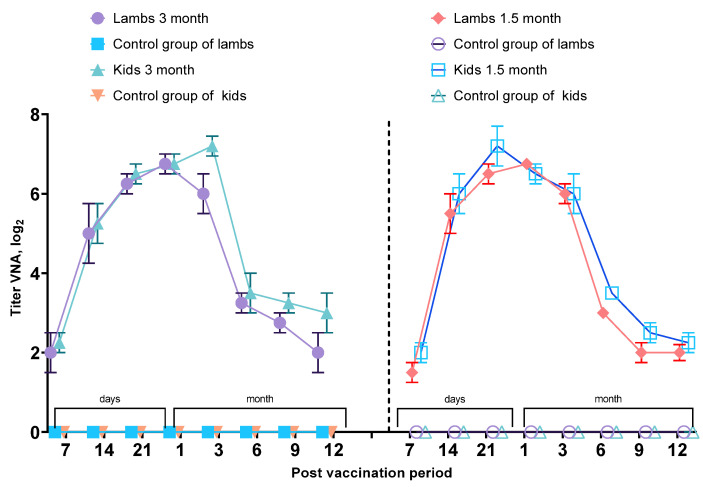
Virus-neutralizing antibody activity in vaccinated and control young sheep and goats following a single vaccination.

Following subcutaneous administration of the experimental vaccine at a dose of 1.0 × 10^3.0^ TCID_50_/mL to 1.5 and 3 months, VNA were detected on day 14 post-vaccination in 80% of immunized 1.5-month-old lambs and kids and in 66% of 3-month-old animals, with mean titers ranging from 3.0 to 5.0 log_2_. By day 21 post-immunization, all vaccinated animals (100%) had developed protective immunity against the PPR virus, with VNA titers ranging from 4.0 to 7.0 log_2_. Mean antibody titers continued to rise, reaching approximately 7.6 log_2_ by day 30, regardless of the animals’ age. However, in 1.5-month-old young animals, a decline in antibody levels was observed starting from week 14 post-vaccination, with VNA titers dropping below 3.0 log_2_ by week 18. A similar gradual decrease in VNA titers was observed in 3-month-old lambs and kids beginning at week 18. By the end of the study (6 months post-vaccination), antibody titers in the 3-month-old group declined to 2.0–3.0 log_2_. These results indicate that, although early protective immunity was achieved by day 21 post-vaccination, the duration of the humoral immune response in young animals was limited and showed a gradual decline over time. Detailed results on virus-neutralizing antibody titers in young animals are presented in Figure 4. In vaccinated 1.5- and 3-month-old lambs and kids, VNA titers increased significantly during the early post-vaccination period (*p* < 0.05), reached peak levels by day 21–30, and then gradually declined over time. No virus-neutralizing antibodies were detected in control animals throughout the study period.

All serum samples from both young and adult sheep and goats prior to immunization were seronegative (S/N > 150%). Within 14 days post-vaccination, the S/N percentage in 1.5-month-old lambs decreased to 85%. Protective antibody levels (ELISA positive) were detected in 50% of the lambs, while the remaining 50% yielded equivocal results (S/N 50–60%). In 1.5-month-old kids, the S/N percentage dropped to 75%; among them, 50% had protective antibody levels, 25% showed values above the threshold (S/N > 60%), and 25% had doubtful results.

In 3-month-old lambs, 14 days after vaccination, the S/N decreased to 78%: 50% tested positive for protective antibodies, 25% remained above the threshold, and 25% showed doubtful ELISA results. In 3-month-old kids, the S/N decreased to 70%, with 75% demonstrating protective antibody levels and 25% showing doubtful results.

**Figure 5:**
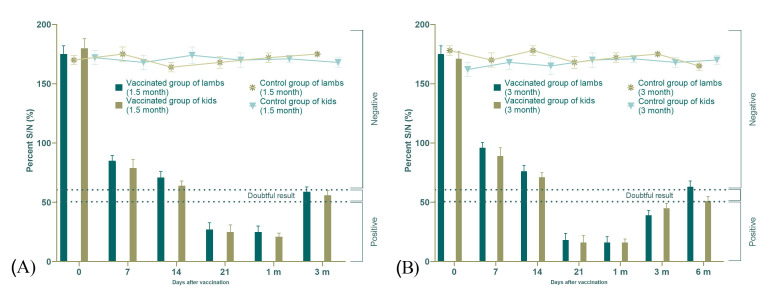
Evolution of the mean percentage inhibition (±standard deviation) in vaccinated and control lamb and kid groups following a single vaccination. (**A**)—Antibody levels against PPR virus in 1.5-month-old lambs and kids; (**B**)—Antibody levels against PPR virus in 3-month-old lambs and kids. Bar charts represent vaccinated animals, while line graphs represent control animals.

A marked decline in S/N values was observed by day 21 post-vaccination—down to 27% in 1.5-month-old and 16% in 3-month-old young animals; at this point, all vaccinated animals (100%) were protected against the PPR virus (Figure 5). The lowest mean S/N percentages were recorded on day 30: approximately 18% in 1.5-month-olds and about 10% in 3-month-olds. However, beginning from week 14 post-vaccination, an increase in S/N% was noted in 1.5-month-old lambs and kids. By week 18, the S/N percentage rose to ~135%, indicating that antibody titers in all these animals had declined below the protective threshold (S/N > 60%). In 3-month-old lambs and kids, the S/N percentages at week 18 were approximately 57% and 43%, respectively. By the end of the experiment (6 months post-vaccination), antibody titers had fallen below the protective level (S/N > 60%) in 75% of the 3-month-old lambs and 50% of the 3-month-old kids, with one kid showing a doubtful ELISA result. The detailed dynamics of S/N% changes are presented in Figure 5. Similarly, S/N values in vaccinated young animals decreased significantly during the early post-vaccination period (*p* < 0.05), consistent with seroconversion, and subsequently increased over time, reflecting a gradual decline in humoral immunity. In contrast, control animals remained seronegative throughout the observation period.

Thus, it was established that a single immunization with the Nigeria 75/1 strain-based vaccine in young lambs and kids (1.5 and 3 months old) induces the production of virus-neutralizing antibodies by day 14 post-vaccination, providing protection against the PPR virus. The humoral immune response in the young animals persisted until approximately week 17–18 post-vaccination in the 1.5-month-old group, and up to 6 months in the 3-month-old group.

### 3.4. Duration of Immunity in Adult Animals for up to 36 Months

**Figure 6:**
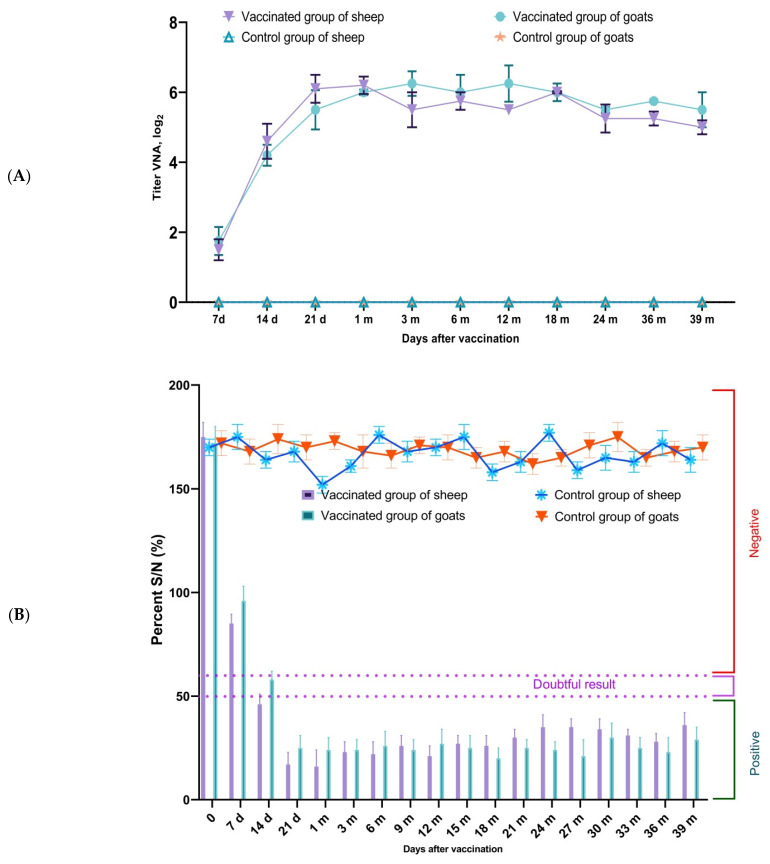
Evaluation of the immune status in adult sheep and goats immunized with a vaccine against PPR.

Evaluation of the duration of immunity in sheep and goats vaccinated with the experimental vaccine showed that even 36 months post-immunization, VNA against the PPRV were still detectable in the blood serum of vaccinated animals at titers of 1:8–1:64 in sheep and 1:16–1:64 in goats (Figure 6A). At this time point, all vaccinated sheep and goats retained humoral immunity to the PPRV. The percentage of competitive inhibition in the ELISA (S/N) at 36 months post-vaccination was approximately 28% in sheep and 23% in goats (Figure 6B). These findings demonstrate sustained humoral immunity in adult animals for up to 36 months following a single immunization. The long-term humoral response remained detectable in both adult sheep and goats throughout the observation period, and the VNA and ELISA results showed comparable trends in the two species.

Administration of the vaccine at an increased dose (10^5.0^ TCID_50_/mL) in young sheep and goats did not result in any deviations from physiological norms, confirming the safety of the developed vaccine. All animals remained clinically healthy; no side effects were observed, except for minor local reactions in the form of swelling (up to ~2.0 × 2.0 cm), which resolved by days 4–5 without an increase in body temperature.

It should be noted that the assessment of safety was based on clinical observations, and no pathological or histopathological examinations were performed. Thus, the developed PPR vaccine demonstrated a favorable safety profile and low reactogenicity in young and adult animals under the conditions of this study.

### 3.5. Clinical Condition of Vaccinated Animals After Challenge Infection

According to the obtained results and taking into account bioethical considerations, the challenge infection of young animals aged 1.5 and 3 months was carried out only on day 21 after immunization. Planned challenge infections at 12, 24, and 36 months were not conducted due to a decline in antibody levels to suboptimal values, and to avoid potential welfare risks to the animals, in accordance with ethical standards.

**Figure 7:**
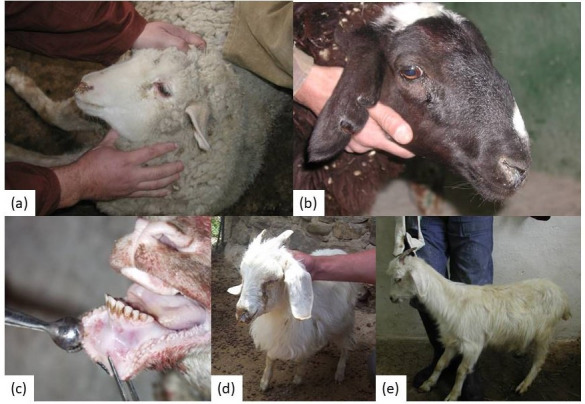
Clinical manifestations of PPR in unvaccinated animals: (**a**,**b**) ocular and nasal discharges in sheep; (**c**) gingival hyperemia in a goat; (**d**,**e**) clinical signs in goats, including ocular and nasal discharges and diarrhea.

**Figure 8:**
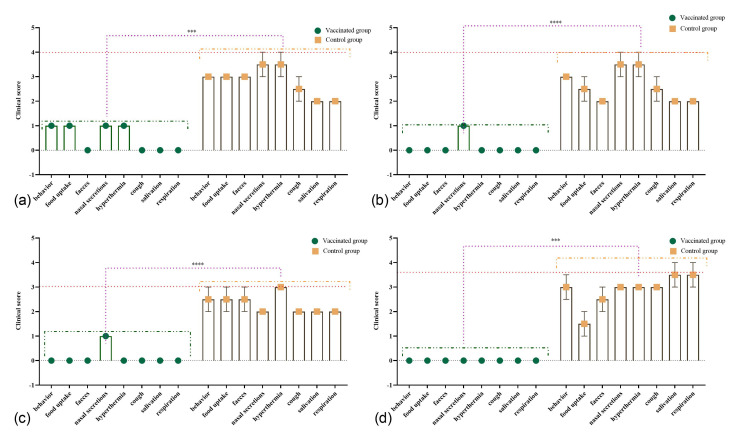
Evaluation of clinical signs in vaccinated and unvaccinated sheep after challenge with virulent PPRV: (**a**–**d**)—dynamics of total clinical score in vaccinated (3 animals per group, *n* = 18) and control (2 animals per group, *n* = 12) sheep and goats following challenge at 21 days (**a**), 12 months (**b**), 24 months (**c**), and 36 months (**d**) post-vaccination. The dashed line indicates the upper threshold of the clinical score. Data are presented as mean ± standard error. *** and **** indicates a significant difference in cumulative clinical scores between the vaccinated and control groups. *p* < 0.0001. Note: Clinical scores are presented as cumulative group scores obtained by summing the values assigned to each evaluated clinical parameter.

In unvaccinated control animals challenged with a virulent PPRV strain, typical clinical manifestations of PPR were observed, including ocular and nasal discharge, gingival hyperemia, general depression, and impaired general condition (Figure 7). In contrast, in a portion of vaccinated animals challenged with a virulent PPRV strain 21 days post-vaccination (vaccinated group), mild clinical signs of the disease were observed (Figure 8). During the symptomatic peak, these animals exhibited mild depression, reduced appetite, lacrimation, serous-mucous nasal discharge, and a slight increase in rectal temperature. Each of these signs was scored as 1 point according to the clinical scoring system; therefore, the cumulative clinical score in individual animals did not exceed 4, whereas in the unvaccinated control group it reached up to 22.5. Rectal temperature was slightly elevated but did not exceed 40.5 °C in adult sheep and 41.2 °C in adult goats, with hyperthermia persisting for up to 4 days with minor fluctuations. In 1.5-month-old lambs, body temperature in one animal fluctuated for 2–3 days, reaching 40.8 °C, while in 3-month-old animals it did not exceed 40.5 °C. Local reactions at the vaccination site (approximately 0.8 × 1.3 cm) resolved spontaneously within 5 days.

In vaccinated sheep and goats challenged at 12 and 24 months post-vaccination, no clinical signs of PPR or other pathologies were observed throughout the observation period, and rectal temperatures remained within physiological limits. Minor local swellings (~0.8 × 1.5 cm) were recorded at the injection site and were scored as 1 point according to the clinical scoring system; these resolved completely within 4–6 days. By comparison, the cumulative clinical score in the unvaccinated control group reached 21 and 18.5 following challenge at 12 and 24 months, respectively.

In vaccinated sheep and goats challenged 36 months after immunization, no clinical signs were observed during the post-infection observation period. Rectal temperature remained within physiological limits, and no local reactions or other abnormalities were recorded. The cumulative clinical score remained 0 (Figure 8), in contrast to the unvaccinated control group, in which it reached 23, indicating a markedly reduced severity of clinical manifestations in vaccinated animals following challenge infection. Statistical analysis of cumulative clinical scores demonstrated a significant reduction in disease severity in vaccinated animals compared with unvaccinated controls across the evaluated challenge periods (two-way ANOVA, *p* < 0.0001; Figure 8). These findings confirm that vaccination significantly reduced the severity of clinical manifestations after challenge infection.

In unvaccinated sheep and goats (control group) challenged with a virulent PPRV strain at 21 days, 12 months, 24 months, and 36 months, clinical signs of disease appeared on days 3–4 post-infection and peaked by days 7–9. The clinical symptoms in control animals were characterized by depression, refusal to eat, ocular and nasal discharges, gingival hyperemia, as well as the development of local reactions measuring 1.0–2.2 cm in diameter (Figure 7). According to the clinical scoring system, the cumulative clinical score in control animals reached 22.5 after challenge at 21 days post-vaccination, 21 after challenge at 12 months, 18.5 after challenge at 24 months, and 23 after challenge at 36 months (Figure 8). Vaccine efficacy, expressed as the proportion of clinically protected animals, was also significantly higher in the vaccinated groups than in the control groups, as confirmed by the one-sided Fisher’s exact test.

More specifically, after challenge at 21 days post-vaccination, the highest scores in control animals were associated with hyperthermia and nasal discharge (up to 3.5 points each), whereas behavioral depression, reduced feed intake, and fecal alterations each reached 3 points; cough, salivation, and respiratory signs were less pronounced (approximately 2.0–2.5 points). After challenge at 12 months, hyperthermia and nasal discharge again represented the most prominent signs (up to 3.5 points each), while behavioral changes, reduced feed intake, fecal disturbances, cough, salivation, and respiratory involvement ranged from 2.0 to 3.0 points. Following challenge at 24 months, hyperthermia reached 3 points, whereas behavioral depression, reduced feed intake, and fecal changes were approximately 2.5 points each; nasal discharge, cough, salivation, and respiratory signs were scored at approximately 2 points. At 36 months post-vaccination, the most pronounced signs in the control group were salivation and respiratory manifestations (up to 3.5 points each), together with hyperthermia, nasal discharge, and cough (approximately 3 points), while behavioral depression, fecal changes, and reduced feed intake ranged from 1.5 to 3 points.

**Figure 9:**
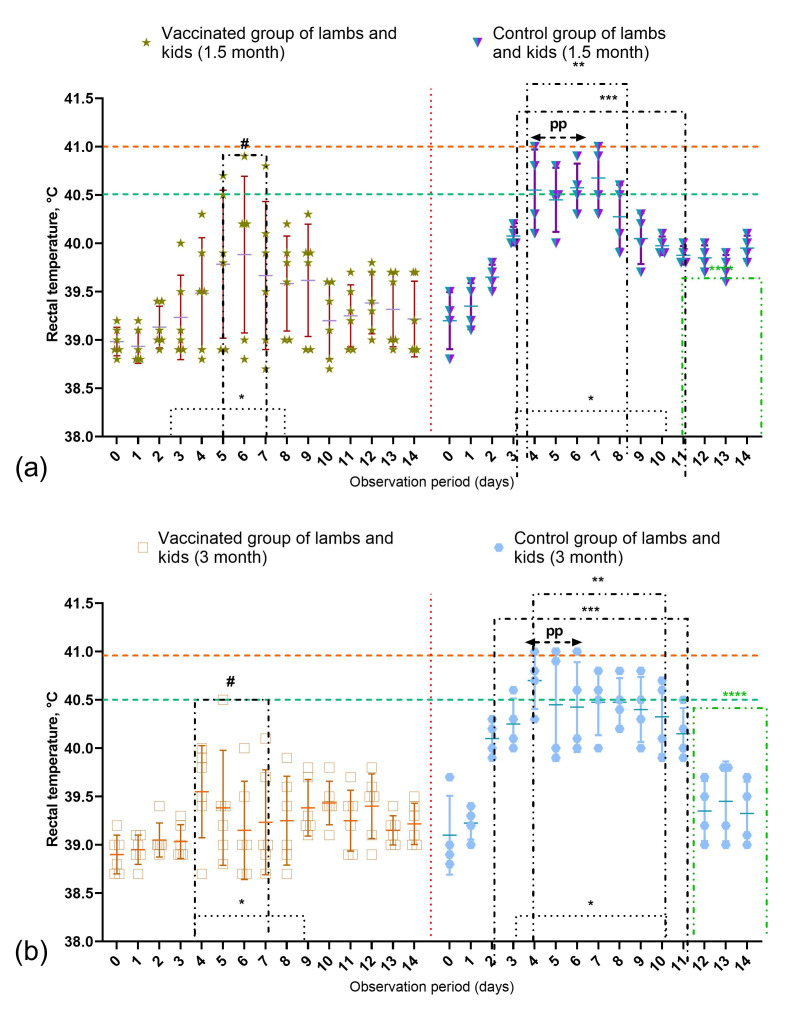

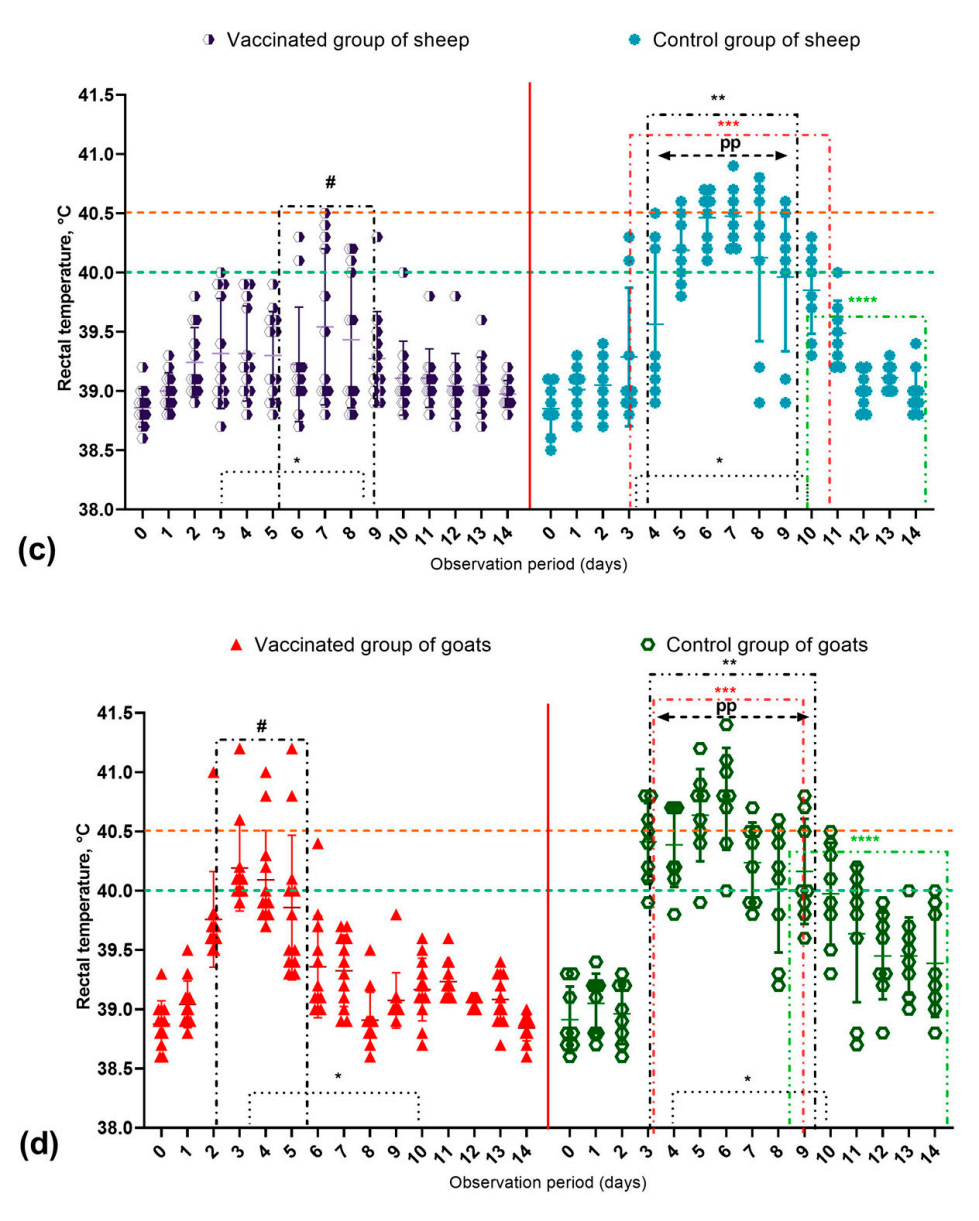
Dynamics of rectal temperature in immunized and control groups of sheep and goats following challenge with virulent PPRV strain: (**a**)—young lambs and kids aged 1.5 months (*n* = 6 vaccinated animals (3 per species), *n* = 4 unvaccinated animals (2 per species)); (**b**)—young lambs and kids aged 3 months (*n* = 6 vaccinated (3 per species), *n* = 4 unvaccinated (2 per species)); (**c**)—adult sheep and (**d**)—goats (*n* = 24 vaccinated (12 per species), *n* = 16 unvaccinated (8 per species)). Vaccinated group—vaccinated sheep and goats challenged with an epizootic strain of PPRV; Control group—unvaccinated sheep and goats challenged with the same strain. The dashed line indicates the upper physiological limit of rectal temperature in sheep. Symbols: (#)—short-term (2-4 day) febrile response in vaccinated animals after challenge; (*)—duration of local reactions in vaccinated and unvaccinated animals after challenge with “Kentau-7” strain; (pp)—pyrexia peak in unvaccinated animals post-challenge; (**)—onset of clinical symptoms in unvaccinated sheep; (***)—duration of elevated rectal temperature in vaccinated animals; (****)—beginning of recovery in infected sheep from day 10 post-infection.

In control animals, fever generally persisted for 5–6 days, with the highest rectal temperatures observed around day 6 post-infection. In two goats challenged at 12 and 24 months, peak hyperthermia reached 41.3 °C, corresponding to 4 points in the clinical scoring system, after which body temperature gradually declined (Figure 9). Surviving control animals were isolated and treated with antibiotics to prevent secondary bacterial complications. Rectal temperature changes were less pronounced and of shorter duration in vaccinated animals than in unvaccinated controls.

Thus, the conducted studies demonstrated that a single immunization of sheep with the PPR vaccine at a dose of 1.0 × 10^3.0^ TCID_50_/mL induces protective immune responses as early as 21 days post-vaccination. In adult animals, these responses were maintained over an extended period, whereas in young animals they persisted for a limited time before gradually declining.

### 3.6. Virus Persistence in Animals

PCR analysis of nasal swab samples collected from all vaccinated animals on days 1–14 post-vaccination yielded negative results, with no detectable PPRV RNA in any samples. Following challenge with the virulent strain “Kentau-7”, PPRV RNA was also not detected in any blood or swab samples from immunized sheep and goats.

**Figure 10:**
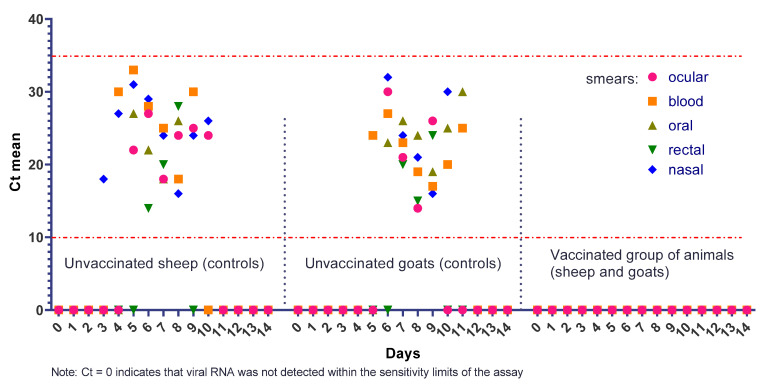
Detection of viral RNA in blood and swab samples from vaccinated and control (unvaccinated) sheep and goats after infection: The dashed lines indicate the assay cut-off values.

At the same time, viral RNA of PPRV was detected in all types of samples collected from unvaccinated control animals (Figure 10). In unvaccinated sheep (control group), viral RNA appeared in nasal swabs as early as day 3 post-infection. In the control group goats that had not been vaccinated, viral RNA was detected in blood samples on day 5, and by day 6 it was found in blood, saliva, ocular, and nasal swabs, indicating systemic viral dissemination. On day 4 post-infection, viral RNA was detected in the blood of 9 out of 12 sheep and 8 out of 12 goats. From day 5 onwards, viral RNA was identified in conjunctival swabs (in 7/12 sheep and 9/12 goats), as well as in oral swabs (in 10/12 sheep and 8/12 goats). Between days 5 and 9, PPRV RNA was also detected in rectal swabs from 4 out of 12 infected sheep and 5 out of 12 goats. PCR-positive samples were detected only in unvaccinated control animals, whereas all samples from vaccinated animals remained negative. These findings support the conclusion that vaccination prevented detectable viremia and viral shedding under the conditions of this study.

## 4. Discussion

Control of peste des petits ruminants (PPR) is primarily achieved through the use of live attenuated vaccines, which provide long-lasting, often lifelong immunity. In the past, when a homologous PPR vaccine was unavailable, a heterologous live attenuated rinderpest vaccine was used to combat the infection [11]. However, the use of heterologous vaccines was later prohibited, as it interfered with serological surveillance in rinderpest-free zones and posed a risk of exposure to the live rinderpest virus [12].

Currently, several PPR vaccines have been developed based on attenuated field virus isolates obtained through serial passages in cell cultures. Four vaccine strains—PPRV/Nigeria/75, PPRV/Sungri/96, PPRV/Arasur/87, and PPRV/Coimbatore/97—are licensed for field use, with commercial formulations based on Nigeria/75 and Sungri/96 strains being widely available [1]. Over the past three decades, various PPRV isolates, including Nigeria 75/1 and Sungri/96, have been successfully attenuated through serial passages in Vero cells and have demonstrated high efficacy in protecting sheep and goats against virulent strains of the virus [28]. Shortly after the first isolation of PPRV in tissue cultures during the 1960s, attempts were made to develop a live attenuated vaccine, but significant success was not achieved until the late 1980s [29,30].

The first homologous vaccine against PPR was developed using the attenuated strain PPRV Nigeria 75/1, marking a significant milestone that allowed for the discontinuation of previous practices involving the use of heterologous rinderpest vaccines to combat PPR. Between 1989 and 1996, several large-scale field trials involving more than 98,000 sheep and goats confirmed the vaccine’s safety (including the absence of abortions in pregnant animals) and demonstrated that protective antibody levels were maintained for up to three years. Furthermore, due to the strong cross-protection between PPRV and RPV, the Nigeria 75/1 vaccine also conferred protection of small ruminants against rinderpest: immunized goats withstood challenge with virulent RPV and did not transmit the infection to susceptible animals [30].

Currently, the attenuated vaccine based on the Nigeria 75/1 strain (genetic lineage II) is produced on a large scale and widely used in all countries of Africa, the Middle East and Asia [13,28]. Although Nigeria 75/1 belongs to lineage II of the PPRV, there is evidence of its successful use in controlling outbreaks caused by lineage IV strains in China (2007, 2013) [31] and Morocco (2008) [32]. Moreover, studies have demonstrated that goats immunized with the Nigeria 75/1 vaccine are protected against virulent Côte d’Ivoire strains (lineage I) [30,33], the virulent Morocco/2008 strain (lineage IV) [32], and the virulent Ghana/78 strain (lineage II). Hodgson et al. showed that the Nigeria 75/1-based vaccine provides broad cross-protection against all four genetic lineages of PPRV [34]. Thus, it is evident that the homologous Nigeria 75/1 strain can be effectively used for PPR control regardless of the circulating viral lineage.

According to the latest data from FAO and its partners, just over 1.2 thousand PPR outbreaks were reported globally in 2019, compared to more than 3.5 thousand outbreaks in 2015. During this period (2015–2019), the disease was most frequently observed in two severely affected regions—Asia and Africa. In recent years (2020–2021), the total number of PPR outbreaks worldwide has significantly declined; however, the geographic range of the infection and the scale of its spread remain extensive.

In Kazakhstan, preventive vaccination against PPR is carried out only in high-risk areas, with the aim of preventing the introduction of the infection from neighboring countries. Until 2015, the veterinary service of the Republic of Kazakhstan, in an effort to prevent the incursion of PPR, annually conducted planned vaccination of high-risk sheep and goats using a live attenuated vaccine based on the G-45MK strain (produced by the RIBSP). This vaccine provided effective immunity against PPR lasting up to one year in vaccinated animals.

When developing prophylactic preparations, particular attention is paid to their safety and immunobiological properties. Our experimental studies have demonstrated that the developed homologous PPR vaccine is capable of inducing a strong humoral immune response associated with protection in vaccinated animals against virulent PPR virus for up to 36 months following a single immunization. Notably, the level of protective immunity established by day 21 post-vaccination persisted for 36 months without significant decline (statistically insignificant fluctuations, *p* > 0.05).

Previously, we demonstrated the effectiveness of a combined vaccine against PPR (strain Nigeria 75/1, GenBank: KY628761.1) and sheep pox (strain NISKhI, GenBank: AY077834.1) in fine-wool Kazakh breed sheep [35]. In particular, it was shown that the vaccine is capable of inducing a stable protective immunity for up to 12 months in animals aged 6 to 12 months. Of particular interest is the immune response in young animals, as the literature lacks sufficient data on the reaction of lambs and kids to PPR vaccination and on the duration of their post-vaccination immunity.

In this regard, we conducted separate studies to evaluate the safety and immunogenicity of the vaccine in young animals aged 1.5 and 3 months. The results obtained indicate that the experimental PPR vaccine was well tolerated in lambs and kids of the specified ages, with no clinically observable adverse effects. Moreover, the humoral immunity induced by a single vaccination (dose 1.0 × 10^3.0^ TCID_50_/mL) persisted in 1.5 month-old animals until approximately the 18th week, and in 3-month-old animals—for approximately 6 months.

Young animals maintain immunogenicity for a shorter period because their immune system is still immature and unable to mount a fully developed and sustained immune response, relying initially on temporary maternal antibodies. This is due to the fact that both the innate and adaptive immune systems require time to fully mature: T-cell development, antibody production, and other immune components are completed only later. However, in the present study, lambs and kids were initially seronegative and lacked detectable maternal antibodies against PPRV, making them dependent on post-vaccination immunity.

It was observed that in young animals (1.5 and 3 months old), the formation of humoral immunity occurred more rapidly than in adults. However, the duration of antibody persistence in young animals was limited: in 1.5-month-old kids and lambs, antibodies were detected up to approximately the 18th week post-vaccination, whereas in 3-month-old animals, antibodies persisted for about 6 months. This indicates the importance of considering the age of animals when planning vaccination schedules and the potential need for revaccination to extend protective immunity. Furthermore, it was noted that the level of specific antibodies in goats was higher compared to sheep, which is presumably due to species-specific features of the immune response and host susceptibility [36]. According to the literature, *Morbillivirus* infection is characterized by pronounced immunosuppression accompanied by the activation of a robust virus-specific immune response. The protective humoral and cellular responses are directed primarily against the H, F, and N proteins. The envelope glycoproteins H and F induce protective neutralizing antibodies, whereas the N protein elicits a strong cell-mediated immune response, generating a pool of memory T cells. In virulent PPRV infection in goats, the proportions of WC1+ γ/δ T cells and CD14+ monocytes remain unchanged; however, a transient decrease in CD4+ cells and a slight increase in CD8+ cells are observed in naïve animals, indicating activation of the CTL response [3]. Seroprevalence of PPRV varies across species and field conditions: several studies report higher antibody levels in sheep, which is attributed to their higher survival rate [37,38,39]. However, other reports indicate greater susceptibility of goats and, consequently, a higher likelihood of seroconversion [40,41,42]. Species-specific differences in the immune response between goats and sheep are important but remain incompletely understood. One possible explanation could be a more pronounced activation of innate immunity in goats, including higher expression of Toll-like receptors (TLRs) and faster production of proinflammatory cytokines that facilitate the activation of adaptive immunity [43]. Moreover, goats may exhibit more intensive proliferation and differentiation of B-lymphocytes, which could accelerate the production of neutralizing antibodies [44,45]. PPRV utilizes two natural cellular receptors: the signaling lymphocyte activation molecule (SLAM, also known as CD150) and Nectin-4. Differences in the kinetics of T-cell responses, including the activation of Th1/Th2 populations, may also play a role. These hypotheses require further comparative immunological studies to establish their significance in conferring protection against PPR [45,46].

Additionally, trials were conducted on adult animals and data on pregnant animals were obtained within the framework of previously published studies to comprehensively assess the immunobiological properties of the vaccine. A single immunization of pregnant sheep and goats (dose 1.0 × 10^3.0^ TCID_50_) did not cause any adverse reactions or result in abortions, as reported in earlier work, confirming the safety of the vaccine for pregnant animals. Furthermore, administration of a higher vaccine dose did not lead to side effects and demonstrated a favorable safety profile [25]. Additionally, preclinical safety/reactogenicity studies were conducted on laboratory animals—including guinea pigs (n = 6, aged 5–7 weeks, weight 200–350 g) and white mice (n = 15, aged 6–8 weeks, weight 18–20 g)—as well as on fine-wool sheep (n = 6) and goats (n = 6) aged 6–12 months. The results of these studies, previously published in a national scientific journal [47], also confirmed the absence of any toxic or adverse effects of the vaccine.

In addition to the serological findings, the clinical observations and rectal temperature monitoring support the favorable tolerability profile of the vaccine. Vaccinated sheep and goats did not show sustained hyperthermia or marked deterioration in general condition after immunization, indicating good clinical tolerability of the vaccine. By contrast, following challenge with the virulent strain, unvaccinated control animals developed clinical signs and temperature changes consistent with acute PPRV infection, whereas vaccinated animals remained clinically stable. These findings complement the serological data and support the protective effect of vaccination under the conditions of this study.

The real-time RT-PCR results provided additional evidence of vaccine-associated protection. No detectable PPRV RNA was found in blood or swab samples from vaccinated animals after challenge, whereas viral RNA was detected in multiple sample types from unvaccinated controls, indicating systemic dissemination and active virus shedding in susceptible animals. Thus, the molecular findings are consistent with the clinical observations and serological results, and support the conclusion that vaccination markedly reduced detectable viremia and shedding within the observation period and within the sensitivity limits of the assay.

This study has several limitations that should be acknowledged. First, long-term challenge protection was demonstrated only in adult animals, whereas challenge infection was not performed in young animals because antibody levels had declined to suboptimal values and such testing was considered inappropriate for bioethical reasons. Second, the study did not include histopathological assessment or dedicated analyses of cell-mediated immunity, which could provide additional insight into vaccine-induced protection.

Following the completion of the preclinical (experimental) studies described in this work, the developed PPR vaccine underwent subsequent stages of evaluation, including commission trials to assess its physicochemical properties, immunobiological efficacy, and production technology under pilot manufacturing conditions. The vaccine strain has been deposited in the RIBSP collection, and based on the cumulative evidence from preclinical and subsequent evaluations, the vaccine has been officially registered and introduced into veterinary practice in the Republic of Kazakhstan.

## 5. Conclusions

The presented study confirmed that the live attenuated PPR vaccine based on the Nigeria 75/1 strain is well tolerated and highly immunogenic in local populations of sheep and goats of different age groups. A detectable humoral immune response was observed by day 14 post-vaccination, whereas protective efficacy was confirmed by challenge infection on day 21. Long-term immunity was demonstrated in adult animals for up to 36 months, while in young animals the immune response was induced early but persisted for a shorter period. The vaccine has successfully passed all stages of preclinical and clinical trials, based on which it has been officially registered in the Republic of Kazakhstan and recommended for practical use. These findings confirm that the vaccine meets international standards of safety and efficacy and highlight its potential to reduce economic losses in livestock production and support the successful implementation of national PPR control and eradication programs.

## Figures and Tables

**Table 1:** The RT-PCR protocol.

Steps	Temperature, °C	Time
Reverse transcription	45 °C	10 min
DNA polymerase activation	95 °C	10 min
40 cycles of denaturation	95 °C	15 s
Annealing/extension	60 °C	60 s

## Data Availability

The original contributions presented in this study are included in the article. Further inquiries can be directed to the corresponding author.

## Abbreviations

The following abbreviations are used in this manuscript:

CDV	canine distemper virus
CPE	cytopathic effects
DMEM	Dulbecco’s Modified Eagle Medium
MV	measles virus
OIE	Office International des Epizooties
NI	neutralization index
PPR	Peste des petits ruminants
PPRV	Peste des petits ruminants virus
VNA	virus-neutralizing antibody
VNT	virus neutralization test
RIBSP	Research Institute for Biological Safety Problems
RPV	rinderpest virus
WOAH	World Organisation for Animal Health
